# A cryptic Gondwana-forming orogen located in Antarctica

**DOI:** 10.1038/s41598-018-26530-1

**Published:** 2018-05-30

**Authors:** Nathan R. Daczko, Jacqueline A. Halpin, Ian C. W. Fitzsimons, Joanne M. Whittaker

**Affiliations:** 1 ARC Centre of Excellence for Core to Crust Fluid Systems and GEMOC, Department of Earth and Planetary Sciences, Macquarie University, Sydney, NSW 2109 Australia; 2 Institute for Marine and Antarctic Studies, University of Tasmania, Private Bag 129, Hobart, Tasmania 7001 Australia; 3 School of Earth and Planetary Sciences, Curtin University, GPO Box U1987, Perth, WA 6845 Australia

## Abstract

The most poorly exposed and least understood Gondwana-forming orogen lies largely hidden beneath ice in East Antarctica. Called the Kuunga orogen, its interpolation between scattered outcrops is speculative with differing and often contradictory trends proposed, and no consensus on the location of any sutures. While some discount a suture altogether, paleomagnetic data from Indo-Antarctica and Australo-Antarctica do require 3000–5000 km relative displacement during Ediacaran-Cambrian Gondwana amalgamation, suggesting that the Kuunga orogen sutured provinces of broadly Indian versus Australian affinity. Here we use compiled data from detrital zircons offshore of East Antarctica that fingerprint two coastal subglacial basement provinces between 60 and 130°E, one of Indian affinity with dominant ca. 980–900 Ma ages (Indo-Antarctica) and one of Australian affinity with dominant ca. 1190–1140 and ca. 1560 Ma ages (Australo-Antarctica). We combine this offshore compilation with existing and new onshore U-Pb geochronology and previous geophysical interpretations to delimit the Indo-Australo-Antarctic boundary at a prominent geophysical lineament which intersects the coast east of Mirny at ~94°E.

## Introduction

## Delineating Ancient Orogenic Belts

**Figure 1:**
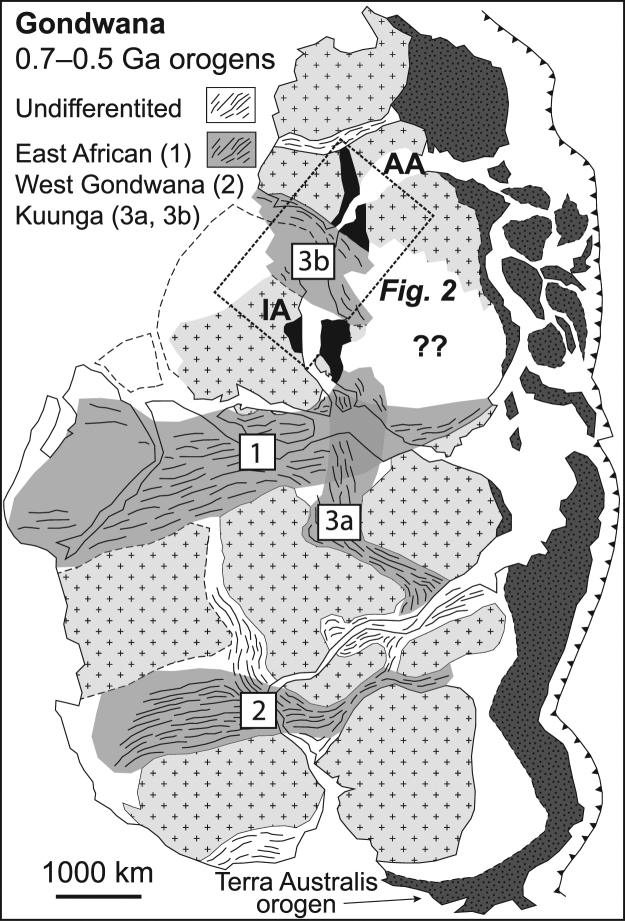
Cratons (>0.9 Ga; light grey-fill) and Pan-African orogens (0.7–0.5 Ga) of Gondwana modified from Fitzsimons^70^. Conjugate margin correlations of Indo-Antarctica (IA) and Australo-Antarctica (AA) are shown (black-fill).

Collisional mountain belts produced by Ediacaran-Cambrian assembly of Gondwana had a profound effect on Earth evolution, with their rapid erosion linked to changes in ocean chemistry, increased atmospheric oxygen, the rise of metazoans and the Cambrian explosion^1–3^. The southern continents preserve numerous 650–500 Ma orogens (Fig. 1), and although the East African orogen is the most likely remnant of a major transgondwanan mountain chain^4^, other mountain belts could have been just as significant^5^, making it difficult to establish precise links between orogenesis, erosion, and environmental change.

The most enigmatic Gondwana-forming orogen lies beneath the ice in East Antarctica. Often called the Kuunga orogen^6^ and interpreted as a collision zone between Indo-Antarctica and Australo-Antarctica^7–11^, this belt was dismantled during Gondwana breakup with much of the Indian segment reworked by collision with Asia, and the remainder mostly concealed by sedimentary basins in India and Australia and an ice sheet in Antarctica. It had a probable length >4000 km, with paleomagnetic data suggesting 3000–5000 km of movement between Indo-Antarctica and Australo-Antarctica between ca. 750 Ma and ca. 500 Ma^12–14^. These palaeomagentic data imply that orogenesis reflects ocean closure at an Ediacaran-Cambrian plate boundary^2,15^.

Sites of ocean closure in modern orogens are ophiolite-decorated sutures. A lack of identified ophiolite, typical arc rocks, or consensus on the location and geometry of any sutures makes it difficult to incorporate the Kuunga orogen into models of Gondwana evolution^16^. Although a prominent inland geophysical lineament at ~100°E was recently interpreted as the suture between Indo-Antarctica and Australo-Antarctica^17^, its age and nature are speculative without supporting outcrop data. Another approach to narrow down suture location is to use the different characteristics of colliding blocks. If Indo-Antarctica and Australo-Antarctica had distinct geological histories, then a suture should juxtapose crust of different protolith age. Indeed this logic has been used to argue that part of the suture between the Indo-Antarctic and Australo-Antarctic plates lies within the southern Prince Charles Mountains^9,18^. This simple model need not hold for complex orogens, for example where final collision was preceded by rifting and transfer of continental ribbons across the intervening ocean, in which case final collision can reunite crust with the same early history. However, this approach can constrain suture location from geochronology of available outcrop even when the suture itself is not exposed, and although it might not be the only suture or the final suture, it is a fundamental boundary and an important first step in determining the overall orogenic architecture in deeply eroded and poorly exposed orogens.

In this paper, we use detrital zircon datasets offshore East Antarctica as a proxy for poorly exposed coastal subglacial geology and to differentiate Indo-Antarctic and Australo-Antarctic age signatures. We then use published and new age data from sparse coastal outcrop to argue that the suture between Indo-Antarctica and Australo-Antarctica lies hundreds of kilometres further west than recent geophysical interpretations. We hypothesise this “India” v “Australia” paleo-plate boundary in Antarctica corresponds to a subglacial fault intersecting the coast at ~94°E.

## Regional Constraints

### U-Pb Age data

Fitzsimons^19^ highlighted an age difference between the Rayner (980–900 Ma) and Wilkes (1330–1130 Ma) provinces of Antarctica, noting they correspond to previously contiguous rocks in India and Australia (Fig. 1). He further argued that their separation by a broad belt of 550–500 Ma metamorphism is consistent with juxtaposition during Ediacaran-Cambrian orogenesis. Although now supported by paleomagnetic data^12^, this model is challenged by local 1380–1020 Ma orthogneiss protoliths in the Rayner province^20,21^, that have been used to argue against any younger plate boundary between the two provinces^22^. The significance of these ages is difficult to assess using the limited data available from sparse outcrop (e.g., compiled by Veevers^23^), but the compilation of detrital ages from offshore sediment (Supplementary Table 1) should be representative of subglacial geology up to 200–500 km from the coast^24^.

**Figure 2:**
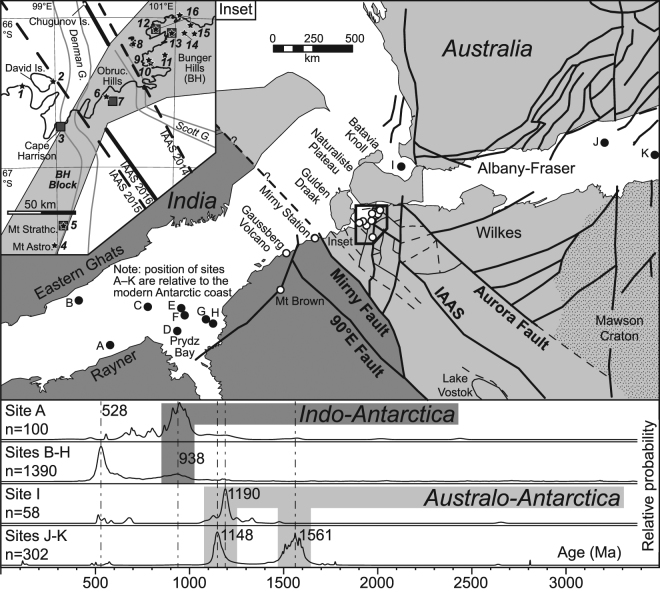
Gondwana reconstruction showing location of: (1) marine sediment samples relative to present-day Antarctic coastline (black filled circles, A-K, Supplementary Table 1), (2) onshore geochronology (white filled circles [main map] and sites 1–16 [inset]), and (3) geophysical lineament IAAS; Indo-Australo-Antarctic Suture^17,33^. Panels at bottom show Gaussian-summation probability density distribution plots of U-Pb age data for offshore detrital zircon grains (Supplementary Table 1). Inset: stars = previously published data (see text for references); grey-filled squares = this study. The geophysically-defined Bunger Hills Block in the inset is from Maritati *et al*.^33^ for spatial reference to our samples. Three previously proposed positions for the IAAS are also shown (see text for discussion).

Figure 2 compiles published U-Pb zircon ages for detritus off the Antarctic coast between 60 and 130°E^25–29^ (Supplementary Table 1). These data confirm that while 550–500 Ma zircon is a minor component of western (site A) and eastern samples (sites J, K), it is significant in intervening samples and abundant in Prydz Bay, matching onshore geology. Equally, eastern samples (sites J, K) contain a 1600–1500 Ma population likely to reflect subglacial equivalents of key elements of South Australian geology (including the Hiltaba Suite granitiods and/or Gawler Range Volcanics^30^) and Terre Adélie geology^31^, whereas ca. 1190–1140 Ma peaks (sites I–K) match dominant ages in the Wilkes province (and formerly contiguous Albany-Fraser orogen of Australia) and ca. 980–900 Ma peaks (sites A–H) match dominant ages in the Rayner province (and formerly contiguous Eastern Ghats belt of India). 1150–1250 Ma zircon grains do occur offshore the Rayner province, consistent with protolith ages reported from outcrop, but their low abundance suggests rocks of this age are volumetrically insignificant in the Prydz Bay catchment. We therefore follow Fitzsimons^19^ in taking ca. 980–900 Ma and 1190–1140 Ma ages as characteristic of Indo-Antarctica and Australo-Antarctica respectively, and use the spatial distribution of these ages to locate a fundamental Ediacaran-Cambrian suture between them.

### Geophysical data

Additional constraints on the location of any sutures in East Antarctica are provided by airborne geophysical data. Aitken *et al*.^17^ defined one lineament (Fig. 2; IAAS) which truncates magnetic trends and follows a prominent change in bedrock topography for >1500 km. It was assumed to intersect the coast near the Scott Glacier (~100.5°E; IAAS 2014 Fig. 2 inset), but Gardner *et al*.^32^ argued it was displaced ~50 km west by later rifting to a location near the Denman Glacier (~99.5°E; IAAS 2015 Fig. 2 inset) and Maritati *et al*.^33^ suggested the IAAS also passes through Chugunov Island, ~100 km off the coast (IAAS 2016 Fig. 2 inset). Though the break in geophysical signature identified by Aitken *et al*.^17^ is consistent with a major boundary, there is no evidence for it being a suture between Indo-Antarctica and Australo-Antarctica. Scarce coastal outcrop provides the only opportunity to test the geophysical interpretation.

## Geochronology Results

Six samples (Fig. 2 inset) collected by J.W. Sheraton and R.J. Tingey in 1986 were selected for zircon geochronology (SHRIMP II, Curtin University) and/or *in situ* monazite geochronology (LA-ICPMS, University of Tasmania; supporting figures and full tabulation of data is provided in supplementary files). The four-digit sample identifiers used here all have the prefix 8628.

**Figure 3:**
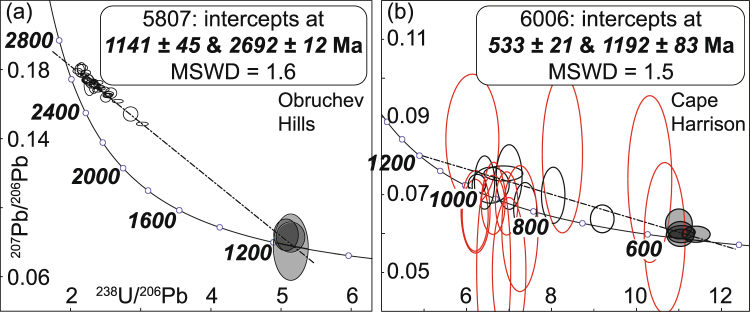
Tera-Wasserberg plots of SHRIMP U-Pb zircon data (2σ error ellipses) and upper and lower intercept ages (95% confidence) calculated from discordia regressions (dash-dot lines). Filled ellipses are analyses of young rims. (**a**) Tonalitic gneiss from Obruchev Hills (5807, site 7). (**b**) Granitic gneiss from Cape Harrison (6006, site 3; red unfilled ellipses excluded from regression due to high common Pb).

Zircon grains (~100–300 μm) from meta-tonalite sample 5807 (Obruchev Hills, site 7), a sample dated previously by conventional bulk zircon analyses^34^, contain bright- to dark-CL oscillatory zoned cores, thick dark-CL mantles and narrow bright-CL oscillatory zoned low-U rims, with an outer moderate-CL overgrowth identified on one grain (#18; Supplementary Figure 1). Thirty-five U-Pb spot analyses were made on 26 grains. All core, mantle and rim domains (n = 32) yield a well-defined discordant data trend with ^207^Pb/^206^Pb dates of ca. 2660–2320 Ma, but three analyses of the thick overgrowth on grain 18 give younger near-concordant ^206^Pb/^238^U dates of ca. 1150 Ma (Fig. 3a). An error-weighted regression of all data yields upper and lower intercepts of 2692 ± 12 and 1141 ± 45 Ma (n = 35; mean square of weighted deviates [MSWD] = 1.6), interpreted as the age of the tonalite protolith and high-grade metamorphism, respectively.

Zircon grains (~150–350 μm) from meta-granite sample 6006 (Cape Harrison, site 3) contain oscillatory zoned cores that range from bright to dark in CL response, with narrow dark-CL rims. Twenty-four analyses of 20 grains show that core domains (n = 20) record a spread of discordant data with scattered ^206^Pb/^238^U dates of ca. 1000–550 Ma, while rim domains (n = 4) give near-concordant ^206^Pb/^238^U dates of ca. 550 Ma (Fig. 3b). Closer inspection reveals that cores comprise discrete high (>0.9) and low (<0.7) Th/U populations, and most of the former have high common Pb. An error-weighted regression of data from rims and low Th/U cores (excluding those with >1% common Pb) yields upper and lower intercepts of 1192 ± 83 Ma and 533 ± 21 Ma (n = 11; MSWD = 1.5; Fig. 3b), with the latter interpreted as the age of metamorphism. Given evidence for multiple core populations, the geological meaning of the upper intercept is difficult to interpret.

**Figure 4:**
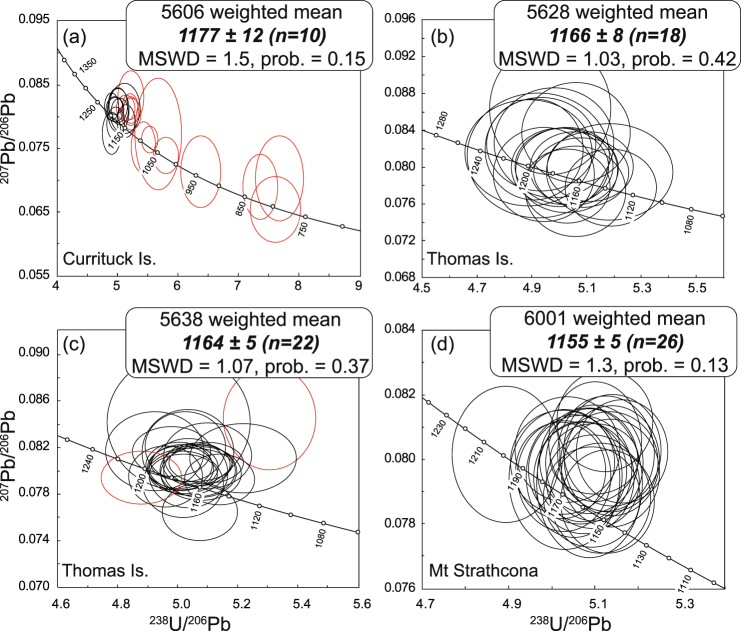
Tera-Wasserberg plots of LA-ICPMS U-Pb monazite data (2σ error ellipses) and weighted mean ^207^Pb corrected ^206^Pb/^238^U ages (95% confidence). Analyses excluded from weighted mean age calculations are shown in red outline. Prob. = probability of fit. (**a**) Metapelite from Currituck Island (5606, site 12). (**b**) Granodioritic gneiss from Thomas Island (5628, site 13), (**c**) metapelite from Thomas Island (5638, site 13). (**d**) felsic paragneiss from Mt Strathcona (6001, site 5).

Analyzed monazite (~60–180 μm) from metapelite sample 5606 (Currituck Island, site 12) occurs in contact with, or enclosed by, garnet, K-feldspar, plagioclase, sillimanite, ilmenite, zircon and quartz. Ten analyses of 4 grains included within garnet give a weighted mean ^207^Pb corrected ^206^Pb/^238^U age of 1177 ± 12 Ma (MSWD = 1.5; Fig. 4a) inferred to date peak granulite-facies metamorphism, and 13 excluded analyses with apparent dates  down to ca. 790 Ma are interpreted to reflect later metamorphism and/or Pb loss. Meta-granodiorite sample 5628 (Thomas Island, site 13) was dated previously by U-Pb zircon^34^, and the monazite grains analyzed in this study (~100–160 μm) occur in contact with, or enclosed by, orthopyroxene, K-feldspar, quartz, and plagioclase. Eighteen analyses of 8 grains have a weighted mean age of 1166 ± 8 Ma (MSWD = 1.03; Fig. 4b) taken as dating granulite metamorphism. Metapelite sample 5638 (Thomas Island, site 13) contains monazite (~20–190 μm) in contact with, or enclosed by, garnet, sillimanite, biotite, quartz, plagioclase and K-feldspar. Twenty-two (out of 24) analyses of 9 grains have a weighted mean age of 1164 ± 5 Ma (MSWD = 1.07; Fig. 4c), interpreted to date the main metamorphic event. Felsic paragneiss sample 6001 (Mt Strathcona, site 5) contains monazite (~30–220 μm) in contact with, or enclosed by, garnet, biotite, quartz, plagioclase, and K-feldspar. All 26 analyses of 14 monazite grains give a weighted mean age of 1155 ± 5 Ma (MSWD = 1.3; Fig. 4d), interpreted as the age of metamorphism.

## Discussion

### Locating a paleo-plate boundary between Indo-Antarctica and Australo-Antarctica

Poor exposure and deep erosion of the Ediacaran to Cambrian Kuunga orogen in India, Australia and Antarctica mask its architecture, and make it difficult to incorporate this orogen into models of Gondwana evolution, during a very important period of change in Earth history. Paleomagnetic data imply that a fundamental boundary between Indo-Antarctica and Australo-Antarctica bisects the ice-covered coast of East Antarctica. Our approach to locate this first-order boundary is to refine the distinct U-Pb age signatures of the key colliding blocks along the Antarctic coast by (i) using offshore detrital zircon datasets as a proxy for subglacial crust and (ii) deriving new U-Pb geochronology from key bedrock locations.

Three of the dated samples were collected from Currituck (5606, site 12) and Thomas (5628, 5638, site 13) islands in the Bunger Hills. These samples yield ca. 1180–1165 Ma monazite ages, confirming the timing of peak metamorphism derived in previous studies (ca. 1190–1150 Ma^34,35^, sites 8–16) and supporting the long-standing notion that this area be considered part of the Wilkes province of Australo-Antarctica^7,9,34,36^. Our 1141 ± 45 Ma age for metamorphism in the Obruchev Hills (site 7) is older than a conventional U-Pb zircon lower intercept age of 1040 ± 53 Ma for the same sample^34^, but a much closer match with metamorphic ages in the Bunger Hills and consistent with a 1142 ± 3.6 Ma age for intrusive charnockite nearby (site 6)^37^. Similarly, ca. 1155 Ma monazite from Mt Strathcona (site 5) and ca. 1190 Ma zircon from Cape Harrison (site 3) indicate that these are also part of the Wilkes province, as are three other samples in this area (sites 4–5) with reported U-Pb zircon ages of ca. 1190–1170 Ma^38^.

In summary, samples from the Bunger Hills (sites 12–13) and locations progressively to the west at Obruchev Hills (5807, site 7), Mt Strathcona (6001, site 5) and Cape Harrison (6006, site 3), all record evidence for growth (or recrystallization) of accessory zircon or monazite at ca. 1190–1140 Ma, an age range characteristic of the onshore Wilkes province and its offshore detritus (Fig. 2). Taken together, these new and published age data indicate that ca. 1190–1140 Ma metamorphism and magmatism of the Wilkes province extend westwards from the Bunger Hills at least as far as Cape Harrison, meaning the Indo-Australo-Antarctic suture must lie further west.

Scarce outcrop between Prydz Bay (~78°E) and Cape Harrison (~99°E) is poorly studied, but recent geochronology from Mt Brown (~86°E), Gaussberg (~89°E) and Mirny station (~93°E) yields 980–920 Ma U-Pb zircon ages^39,40^, characteristic of the onshore Rayner province (e.g., Boger^9^ and references therein) and its offshore detritus (Fig. 2). This suggests that crust of Indian affinity extends at least as far east as Mirny station, a conclusion supported by similar feldspar Pb isotope compositions at Prydz Bay and Mirny^41^.

Archean orthogneiss from Cape Charcot (site 1) and Cambrian syenite from David Island (site 2) are the only dated samples between Cape Harrison and Mirny (Fig. 2 inset), yet these lack Proterozoic zircon characteristic of either Australo-Antarctica and Indo-Antarctica^36^. However, continental fragments (Gulden Draak Knoll, Batavia Knoll and Naturaliste Plateau) that reconstruct to lie adjacent to this part of East Antarctica (Fig. 2) have Archean (2.8 Ga), Mesoproterozoic (1.3–1.1 Ga) and Ediacaran to Cambrian (580–510 Ma) ages^32,42,43^, supporting an Australo-Antarctic affinity for the Cape Charcot region.

Together, these geochronology data place the fundamental boundary between Indo-Antarctica and Australo-Antarctica in a ~250 km stretch of coast between Mirny and Cape Charcot (Fig. 2). Aitken *et al*.^17^ identified an unnamed SSW-trending geophysical lineament east of Mirny station at ~94°E. We name this lineament the Mirny Fault and consider it the most likely coastal location of a boundary between Indo-Antarctica and Australo-Antarctica.

### Implications for a cryptic Gondwana-forming orogen

Our new U-Pb geochronology, combined with regional age data and aerogeophysical interpretations, define a NNW-trending paleo-plate boundary that projects across Gondwana from NE India, past Mirny and toward the interior of Antarctica (Fig. 2). Our interpretation draws attention to the likelihood of a paleo-plate boundary and crust of Australo-Antarctic affinity buried in present-day far NE India that requires further investigation. Along strike, the southern portion of the paleo-plate boundary heads toward Lake Vostok, where a poorly-dated subglacial ancient plate boundary is also interpreted from geophysical data^44^.

Though our research locates the fundamental boundary between Indo-Antarctica and Australo-Antarctica along the East Antarctic coast, the overall orogenic architecture includes a series of sub-parallel NNW-trending subglacial structures between the 90°E and Aurora faults (Fig. 2). Therefore, we infer that multiple structures (possibly sutures) were an important part of this orogenic system during final Gondwana amalgamation. Hf-isotopic data in zircon from Ediacaran to Cambrian granite and other regional geological constraints are consistent with dominantly sinistral strike-slip to transpressional motion within this segment of the orogenic system^43^.

Available paleomagnetic data do not accurately constrain the age of collision between Indo-Antarctica and Australo-Antarctica and final Gondwana amalgamation. This problem may be compounded by the predominance of Cambrian zircon in offshore detritus (c. 528 Ma age peak, Fig. 2) that might not reflect the true age of amalgamation. For example, regional granites reported from Gulden Draak and Batavia knolls^32,43^, East Antarctica^36,45^, and in NE India^46,47^ show limited field or petrographic evidence of deformation, suggesting many are late to post-orogenic and therefore crystallised after the amalgamation of Indo-Antarctica and Australo-Antarctica. Collision is likely to have occurred some time between spatially isolated ca. 550–500 Ma ages across eastern Gondwana^32,42,48–54^ and c. 630–620 Ma biotite recrystallisation ages in the SW Yilgarn Craton^55^. These observations question the tectonic significance of regional metamorphic and zircon Pb-loss ages, such as those presented in this paper, some of which could also be argued to relate to post-orogenic processes during collapse of the Kuunga orogen. The exact timing of collision between Indo-Antarctica and Australo-Antarctica and the timing of orogenic collapse remain open to debate.

Future work in this region should focus on rare outcrop east of Mirny to test the crustal affinity of this stretch of coastline. The identification of an Ediacaran–Cambrian paleo-plate boundary between Indo-Antarctic and Australo-Antarctic crust locates a cryptic Gondwana-forming orogen in East Antarctica. This research will lead to improvements in plate models for the amalgamation of Gondwana and help understand a profound period of change in ocean chemistry, increased atmospheric oxygen, and the Cambrian explosion of life^1–3^.

## Methods

### Zircon sample preparation and SHRIMP U-Pb analyses

Zircon grains from 8628–5807 and 8628–6006 were hand-picked and mounted into a 25-mm diameter epoxy resin disc along with grains of reference zircons BR266 (559 Ma, 909 ppm U^56^) and OGC-1 (3465 Ma^57^) and a fragment of NBS610 glass (used to center the 204Pb peak). The mount was polished to expose the zircon grains and reference materials, then carbon-coated for cathodoluminescence imaging on a TESCAN Mira 3 scanning electron microscope in the John de Laeter Centre, Curtin University. The carbon coat was removed and the mount gold-coated prior to U-Pb isotope analysis on the SHRIMP II sensitive high resolution ion microprobe at the John de Laeter Centre, Curtin University.

Analytical procedures for the Curtin SHRIMP II facility were described by Kennedy and De Laeter^58^ and De Laeter and Kennedy^59^ and are similar to those described by Compston *et al*.^60^ and Williams^61^. A mass-filtered primary beam of O2– ions at 10 keV with 25–30 μm diameter was used to sputter secondary ions from the target material. The primary beam current measured at the mount surface was ~2.0 nA, and the beam was rastered over each analysis site for 3–4 minutes to remove surface contamination before secondary ions were collected in 6 scans through the following masses: 196 (90Zr216O+, 2 seconds), 204 (204Pb+, 10 seconds), 205.5 (background, 10 seconds), 206 (206Pb+, 20 seconds), 207 (207Pb+, 30 seconds), 238 (238U+, 3 seconds), 248 (232Th16O+, 2 seconds) and 254 (238U16O+, 3 seconds). Values of 206Pb/238U in zircons from 8628–5807 and 8628–6006 were calibrated using analyses of reference zircon BR266, assuming a power law relationship between 206Pb+/238U+ and 238U16O+/238U+ and a fixed exponent of 2^62^. External spot-to-spot uncertainty (1σ) in 238U/206Pb values in BR266 over the analytical session was 1.03%. Values of 207Pb/206Pb were monitored using the OGC-1 reference zircon which yielded an error-weighted mean 207Pb/206Pb date (95% confidence) of 3466.3 ± 4.8 Ma for the analytical session, within uncertainty of the reference value (3465.4 Ma).

Data were processed and displayed using the Excel add-ins SQUID 2.50.09.08.06^63^ and Isoplot 3.76.12.02.24^64^. All analyses were corrected for common Pb based on measured 204Pb^60^ and common Pb isotope ratios appropriate for the approximate age of zircon crystallization according to the Stacey and Kramers^65^ model of Pb isotope evolution. This assumes that any common Pb is inherent to the zircon crystal, which appears to be the case here given that common Pb contents vary consistently between different zircon domains. In particular, the highest common Pb contents in 8628–6006 are typically associated with high Th/U cores whereas low Th/U cores and rims mostly have lower levels of common Pb. Uncertainties for individual spot analyses of unknown zircons include errors from counting statistics, errors from the common Pb correction and the U-Pb calibration errors based on reproducibility of U-Pb measurements of the standard, and are quoted at the 1σ level in the Supplementary data tables and figures, but error ellipses in concordia diagrams are plotted at the 2σ level. Uncertainties on discordia upper and lower intercepts are quoted with 95% confidence limits.

### Monazite sample preparation and LA-ICPMS analyses

Monazite grains from samples 8628–5606, 8628–5638, 8628–5628 and 8628–6001were analysed *in situ* in polished blocks mounted in 2-inch round mounts. Monazite grains were identified using a FEI Quanta 600 SEM controlled by an automated software package (Mineral Liberation Analyser), and high resolution, high contrast BSE images (Supplementary Fig. 3) were obtained for individual monazite grains using a Hitachi SU-70 field emission (FE)-SEM at the Central Science Laboratory, University of Tasmania. Further details on sample preparation and *in situ* monazite identification can be found in Halpin *et al*.^66^. U–Pb monazite analyses were performed on an Agilent 7500cs quadrupole ICPMS with a 193 nm Coherent Ar–F gas laser and the Resonetics S155 ablation cell at the University of Tasmania. LA-ICPMS setup and conditions, and monazite data reduction and reproducibility, are described in detail in Halpin *et al*.^66^ and summarised below. Tera-Wasserburg diagrams and weighted mean age calculations (Fig. 4) were made using Isoplot v4.11^64^. Error ellipses on Tera-Wasserburg plots are calculated at the two-sigma level and weighted mean and intercept ages are reported at 95% confidence limits. Full tabulation of monazite isotopic data is presented in Supplementary Table 3.

Each analysis was pre-ablated with 5 laser pulses to remove the surface contamination then the blank gas was analysed for 30 s followed by 30 s of monazite ablation at 5 Hz and ∼2 J/cm^2^ using a spot size of 9 μm; keeping U and Th in the pulse counting mode of detection on the electron multiplier. Elements measured included ^31^P, ^56^Fe, ^89^Y, ^202^Hg, ^204^Pb, ^206^Pb, ^207^Pb, ^208^Pb, ^232^Th and ^238^U with each element being measured sequentially every 0.16 s with longer counting time on the Pb isotopes compared to the other elements. The down hole fractionation, instrument drift and mass bias correction factors for Pb/U and Pb/Th ratios on monazites were calculated using analyses on the in-house primary standard (14971 Monazite) and secondary standard monazite grains (RGL4B and Banaeira) analysed at the beginning of the session and every 15–20 unknowns, using the same spot size and conditions as used on the samples to provide an independent control to assess accuracy and precision. The correction factor for the ^207^Pb/^206^Pb ratio was calculated using 8 analyses of the international glass standard NIST610 analysed throughout analytical session and corrected using the values recommended by Baker *et al*.^67^. All data reduction calculations and error propagations were done within Microsoft Excel® via macros designed at the University of Tasmania and summarised in Halpin *et al*.^66^. ^207^Pb corrected ^206^Pb/^238^U weighted mean age for the secondary monazite standard Banaeira is 507 ± 4 Ma (n = 5, MSWD = 0.50), within error of the reference ages of 507.7 ± 1.3 Ma^68^. ^207^Pb corrected ^206^Pb/^238^U weighted mean age for the secondary monazite standard RGL4b is 1560 ± 13 Ma (n = 5, MSWD = 0.30), within error of the reference age of 1566 ± 3 Ma^69^.

## Electronic supplementary material

Supplementary Figures 1–3

Supplementary Table 1

Supplementary Table 2

Supplementary Table 3
