# Supplementary material for: A cryptic Gondwana-forming orogen located in Antarctica
Source: Sci Rep. 2018 May 30;8:8371. doi: 10.1038/s41598-018-26530-1 (PMC5976760; doi:10.1038/s41598-018-26530-1)

A cryptic Gondwana-forming orogen located in Antarctica

Nathan R. Daczko, Jacqueline A. Halpin, Ian C.W. Fitzsimons and Joanne M. Whittaker

Supplementary Files:

Supplementary Figure 1 – zircon CL images and spot analyses for sample 5807 (pages 2-3 of this document).
Supplementary Figure 2 – zircon CL images and spot analyses for sample 6006 (page 4 of this document).
Supplementary Figure 3 – monazite BSE images and spot analyses for samples 5606, 5638, 5628 and 6001 (page 5 of this document).

Supplementary Table 1 – compilation of offshore zircon data in separate Excel (.xlsx) file.
Supplementary Table 2 – new zircon SHRIMP data in separate Word (.docx) file.
Supplementary Table 3 – new monazite LA-ICPMS data in separate Excel (.xlsx) file.

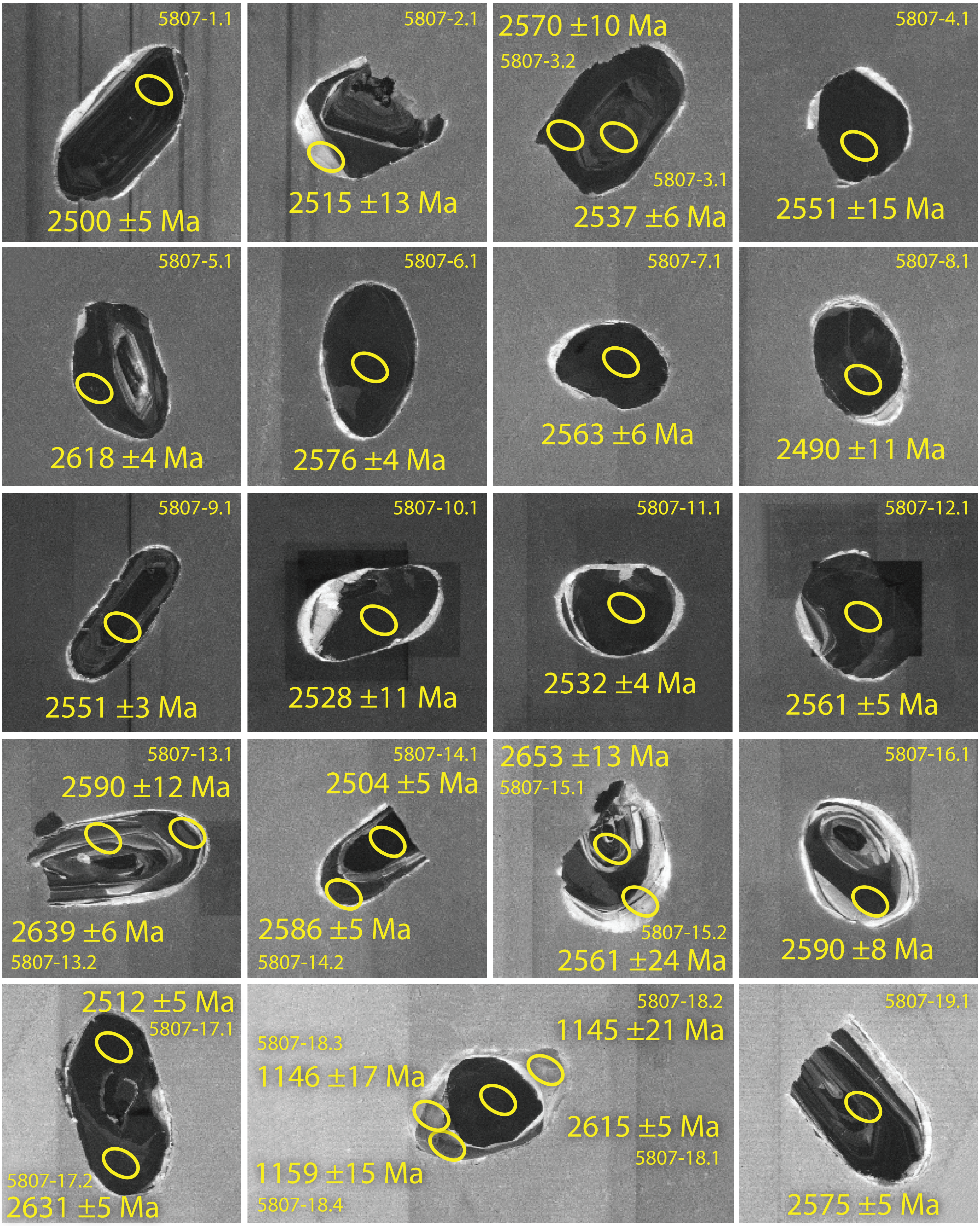
5807-1.1
2500 ±5 Ma
5807-2.1
2515 ±13 Ma
2570 ±10 Ma
5807-3.2
5807-3.1
2537 ±6 Ma
5807-4.1
2551 ±15 Ma
5807-5.1
2618 ±4 Ma
5807-6.1
2576 ±4 Ma
5807-7.1
2563 ±6 Ma
5807-8.1
2490 ±11 Ma
5807-9.1
2551 ±3 Ma
5807-10.1
2528 ±11 Ma
5807-11.1
2532 ±4 Ma
5807-12.1
2561 ±5 Ma
5807-13.1
2590 ±12 Ma
2639 ±6 Ma
5807-13.2
5807-14.1
2504 ±5 Ma
2586 ±5 Ma
5807-14.2
2653 ±13 Ma
5807-15.1
5807-15.2
2561 ±24 Ma
5807-16.1
2590 ±8 Ma
2512 ±5 Ma
5807-17.1
5807-17.2
2631 ±5 Ma
5807-18.2
1145 ±21 Ma
5807-18.3
1146 ±17 Ma
2615 ±5 Ma
5807-18.1
1159 ±15 Ma
5807-18.4
5807-19.1
2575 ±5 Ma

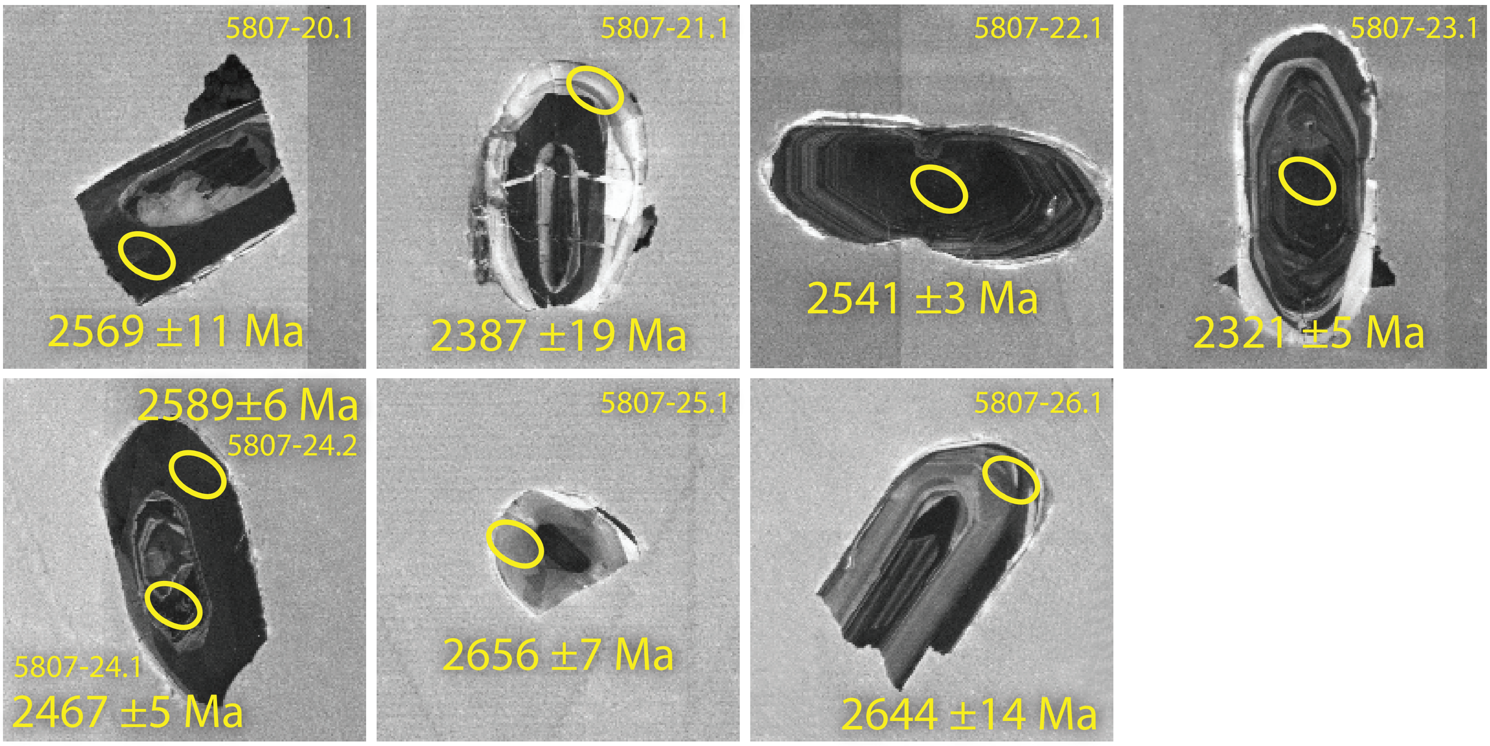

Supplementary Figure 1: Cathodoluminescence (CL) images of all analysed zircon grains from sample 8628-5807, annotated with SHRIMP analysis spot locations, analysis labels, and spot dates with 1σ errors. In most case these are 207Pb/206Pb dates (all dates > 2300 Ma), but the 206Pb/238U date is quoted for anlayses 5807-18.2, 18.3 and 18.4. Note that these dates do not directly reflect the age of zircon crystallization because all anlayses are discordant.

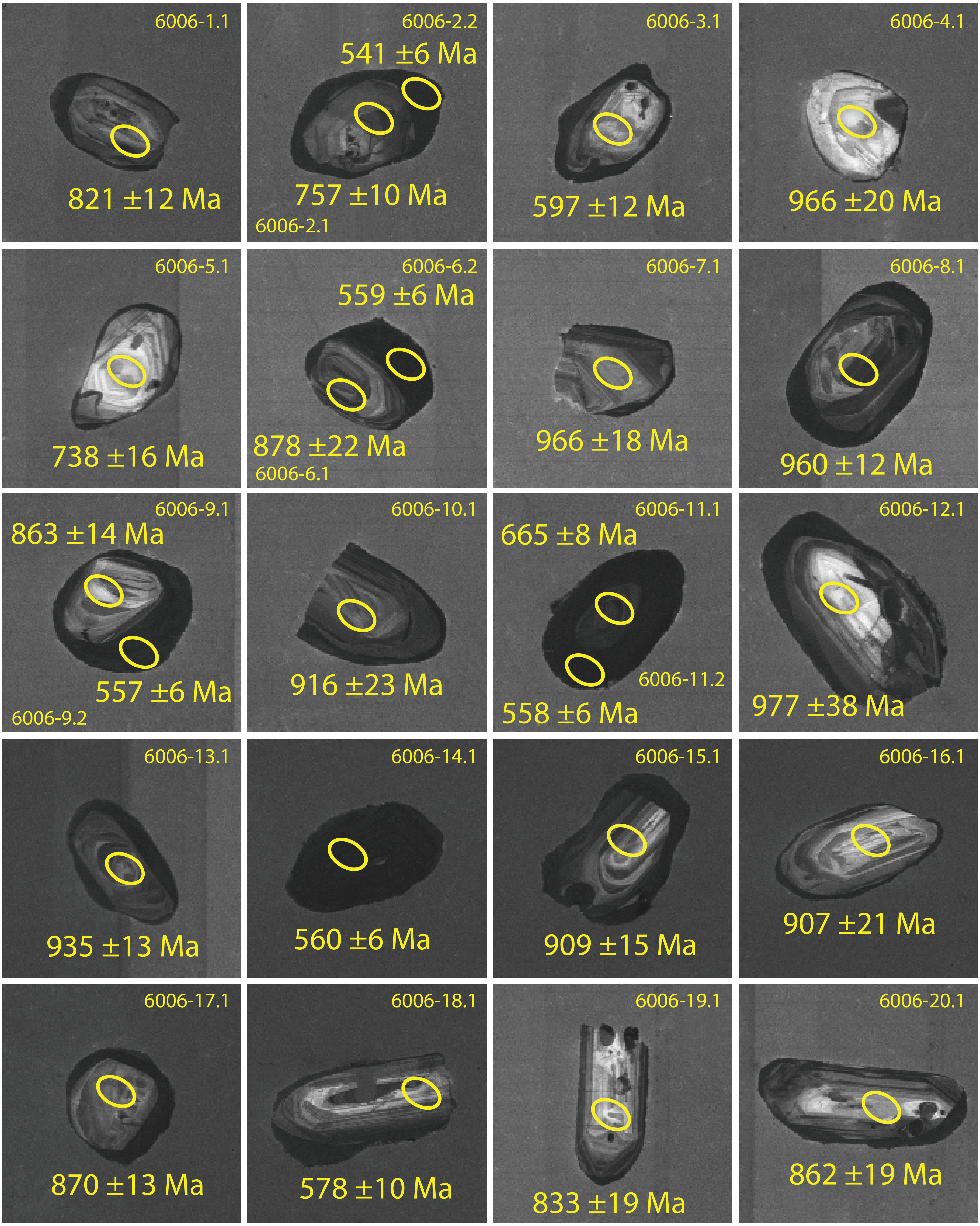

Supplementary Figure 2: Cathodoluminescence (CL) images of all analysed zircon grains from sample 8628-6006, annotated with SHRIMP analysis spot locations, analysis labels, and 206Pb/238U spot dates with 1σ errors. Note that these dates do not directly reflect the age of zircon crystallization because all analyses are discordant.

Supplementary Fig.3. Representative monazite BSE images with individual 207Pb corrected 206Pb/238U ages. Analysis numbers (italics) correspond to Supplementary Table 3.

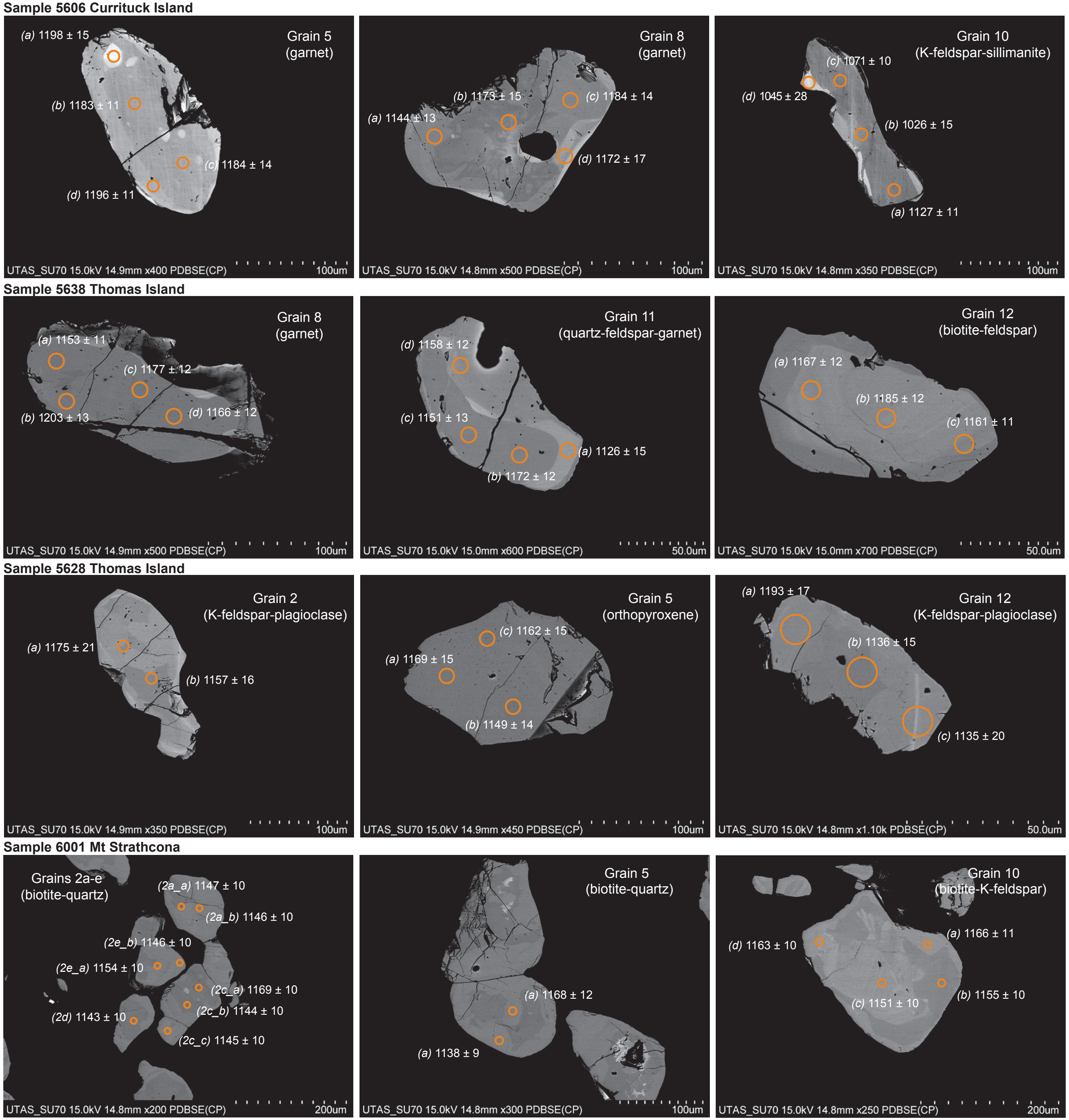

Supplement: Supplementary file 1 — Supplementary Figures 1–3 [file 41598_2018_26530_MOESM1_ESM.pdf]
