# Supplementary material for: A cryptic Gondwana-forming orogen located in Antarctica
Source: Sci Rep. 2018 May 30;8:8371. doi: 10.1038/s41598-018-26530-1 (PMC5976760; doi:10.1038/s41598-018-26530-1)
Supplement: Supplementary file 3 — Supplementary Table 2 [file 41598_2018_26530_MOESM3_ESM.docx]

A cryptic Gondwana-forming orogen located in Antarctica. Nathan R. Daczko, Jacqueline A. Halpin, Ian C.W. Fitzsimons and Joanne M. Whittaker

**Supplementary Table 2a: SHRIMP U-Pb zircon data for 8628-5807**

| Analysis Label | U (ppm) | Th (ppm) | ^232^Th/^238^U | ^206^Pb_c_ (%) | ^238^U/^206^Pb | ±1σ (%) | ^207^Pb/^206^Pb | ±1σ (%) | ^238^U/^206^Pb* 204 corr | ±1σ (%) | ^207^Pb*/^206^Pb* 204 corr | ±1σ (%) | ^238^U/^206^Pb* date (Ma) | ±1σ (Ma) | ^207^Pb*/^206^Pb* date (Ma) | ±1σ (Ma) | Disc (%) |
| --- | --- | --- | --- | --- | --- | --- | --- | --- | --- | --- | --- | --- | --- | --- | --- | --- | --- |
| 5807-1.1 | 1953 | 723 | 0.38 | 0.00 | 2.520 | 1.10 | 0.16424 | 0.29 | 2.520 | 1.10 | 0.16424 | 0.29 | 2155 | 20 | 2500 | 5 | 16 |
| 5807-2.1 | 182 | 69 | 0.39 | 0.09 | 2.566 | 1.36 | 0.16645 | 0.73 | 2.568 | 1.36 | 0.16569 | 0.75 | 2120 | 25 | 2515 | 13 | 18 |
| 5807-3.1 | 314 | 53 | 0.17 | 0.05 | 2.465 | 1.24 | 0.16833 | 0.34 | 2.466 | 1.24 | 0.16794 | 0.35 | 2194 | 23 | 2537 | 6 | 16 |
| 5807-3.2 | 444 | 161 | 0.37 | 0.02 | 2.288 | 1.40 | 0.17140 | 0.62 | 2.289 | 1.40 | 0.17122 | 0.62 | 2337 | 28 | 2570 | 10 | 11 |
| 5807-4.1 | 493 | 193 | 0.40 | 0.03 | 2.319 | 1.18 | 0.16965 | 0.90 | 2.319 | 1.18 | 0.16936 | 0.90 | 2311 | 23 | 2551 | 15 | 11 |
| 5807-5.1 | 540 | 331 | 0.63 | 0.04 | 2.187 | 1.17 | 0.17664 | 0.24 | 2.188 | 1.17 | 0.17627 | 0.25 | 2426 | 24 | 2618 | 4 | 9 |
| 5807-6.1 | 519 | 191 | 0.38 | 0.05 | 2.276 | 1.45 | 0.17229 | 0.25 | 2.277 | 1.45 | 0.17187 | 0.26 | 2347 | 28 | 2576 | 4 | 11 |
| 5807-7.1 | 639 | 390 | 0.63 | 0.05 | 2.327 | 1.15 | 0.17098 | 0.36 | 2.328 | 1.15 | 0.17056 | 0.36 | 2303 | 22 | 2563 | 6 | 12 |
| 5807-8.1 | 422 | 191 | 0.47 | 0.05 | 2.492 | 1.20 | 0.16371 | 0.66 | 2.493 | 1.20 | 0.16325 | 0.66 | 2174 | 22 | 2490 | 11 | 15 |
| 5807-9.1 | 915 | 141 | 0.16 | 0.08 | 2.407 | 1.13 | 0.17006 | 0.19 | 2.409 | 1.13 | 0.16934 | 0.21 | 2239 | 21 | 2551 | 3 | 14 |
| 5807-10.1 | 607 | 376 | 0.64 | 0.03 | 2.391 | 1.16 | 0.16725 | 0.63 | 2.392 | 1.16 | 0.16707 | 0.64 | 2252 | 22 | 2528 | 11 | 13 |
| 5807-11.1 | 556 | 209 | 0.39 | 0.05 | 2.370 | 1.19 | 0.16786 | 0.25 | 2.371 | 1.19 | 0.16740 | 0.26 | 2269 | 23 | 2532 | 4 | 12 |
| 5807-12.1 | 423 | 202 | 0.49 | 0.03 | 2.353 | 1.20 | 0.17063 | 0.28 | 2.354 | 1.20 | 0.17036 | 0.29 | 2282 | 23 | 2561 | 5 | 13 |
| 5807-13.1 | 299 | 116 | 0.40 | 0.03 | 2.244 | 1.24 | 0.17356 | 0.72 | 2.245 | 1.24 | 0.17330 | 0.72 | 2375 | 25 | 2590 | 12 | 10 |
| 5807-13.2 | 243 | 26 | 0.11 | 0.07 | 2.088 | 1.28 | 0.17910 | 0.34 | 2.089 | 1.28 | 0.17851 | 0.36 | 2522 | 27 | 2639 | 6 | 5 |
| 5807-14.1 | 1546 | 393 | 0.26 | 0.02 | 2.532 | 1.10 | 0.16476 | 0.30 | 2.533 | 1.10 | 0.16462 | 0.30 | 2145 | 20 | 2504 | 5 | 17 |
| 5807-14.2 | 343 | 120 | 0.36 | 0.05 | 2.233 | 1.22 | 0.17338 | 0.29 | 2.235 | 1.22 | 0.17296 | 0.30 | 2384 | 24 | 2586 | 5 | 9 |
| 5807-15.1 | 487 | 64 | 0.13 | 0.03 | 2.142 | 1.19 | 0.18034 | 0.76 | 2.143 | 1.19 | 0.18006 | 0.77 | 2469 | 24 | 2653 | 13 | 8 |
| 5807-15.2 | 184 | 78 | 0.44 | 0.35 | 2.253 | 1.37 | 0.17338 | 1.37 | 2.261 | 1.37 | 0.17032 | 1.44 | 2361 | 27 | 2561 | 24 | 9 |
| 5807-16.1 | 509 | 237 | 0.48 | 0.04 | 2.215 | 1.18 | 0.17358 | 0.46 | 2.216 | 1.18 | 0.17327 | 0.46 | 2401 | 24 | 2590 | 8 | 9 |
| 5807-17.1 | 536 | 236 | 0.45 | 0.08 | 2.538 | 1.20 | 0.16607 | 0.28 | 2.540 | 1.20 | 0.16540 | 0.30 | 2140 | 22 | 2512 | 5 | 17 |
| 5807-17.2 | 406 | 162 | 0.41 | 0.06 | 2.204 | 1.23 | 0.17820 | 0.31 | 2.206 | 1.23 | 0.17769 | 0.32 | 2410 | 25 | 2631 | 5 | 10 |
| 5807-18.1 | 507 | 221 | 0.45 | 0.08 | 2.157 | 1.20 | 0.17663 | 0.26 | 2.159 | 1.20 | 0.17594 | 0.27 | 2454 | 24 | 2615 | 5 | 7 |
| 5807-18.2 | 64 | 84 | 1.36 | 2.74 | 5.002 | 1.92 | 0.09989 | 6.38 | 5.142 | 1.99 | 0.07680 | 10.19 | 1145 | 21 | 1116 | 203 | -3 |
| 5807-18.3 | 102 | 44 | 0.45 | 1.39 | 5.068 | 1.60 | 0.09416 | 2.87 | 5.140 | 1.62 | 0.08250 | 4.29 | 1146 | 17 | 1257 | 84 | 10 |
| 5807-18.4 | 178 | 67 | 0.39 | 0.87 | 5.032 | 1.38 | 0.09123 | 2.79 | 5.077 | 1.41 | 0.08391 | 4.44 | 1159 | 15 | 1290 | 86 | 11 |
| 5807-19.1 | 499 | 117 | 0.24 | 0.08 | 2.387 | 1.20 | 0.17249 | 0.28 | 2.388 | 1.20 | 0.17182 | 0.29 | 2254 | 23 | 2575 | 5 | 15 |
| 5807-20.1 | 550 | 288 | 0.54 | 0.10 | 2.323 | 1.19 | 0.17198 | 0.62 | 2.325 | 1.19 | 0.17115 | 0.63 | 2306 | 23 | 2569 | 11 | 12 |
| 5807-21.1 | 141 | 79 | 0.58 | 0.26 | 2.839 | 1.50 | 0.15583 | 1.02 | 2.846 | 1.50 | 0.15366 | 1.11 | 1941 | 25 | 2387 | 19 | 22 |
| 5807-22.1 | 1249 | 292 | 0.24 | 0.01 | 2.423 | 1.13 | 0.16845 | 0.18 | 2.424 | 1.13 | 0.16833 | 0.19 | 2227 | 21 | 2541 | 3 | 15 |
| 5807-23.1 | 737 | 148 | 0.21 | 0.02 | 3.018 | 1.17 | 0.14805 | 0.29 | 3.019 | 1.17 | 0.14786 | 0.29 | 1844 | 19 | 2321 | 5 | 24 |
| 5807-24.1 | 832 | 166 | 0.21 | 0.10 | 2.663 | 1.16 | 0.16193 | 0.25 | 2.666 | 1.16 | 0.16104 | 0.27 | 2053 | 20 | 2467 | 5 | 20 |
| 5807-24.2 | 491 | 168 | 0.35 | 0.13 | 2.223 | 1.24 | 0.17434 | 0.31 | 2.226 | 1.24 | 0.17321 | 0.34 | 2392 | 25 | 2589 | 6 | 9 |
| 5807-25.1 | 181 | 86 | 0.49 | 0.09 | 2.157 | 1.39 | 0.18111 | 0.42 | 2.159 | 1.39 | 0.18030 | 0.45 | 2454 | 28 | 2656 | 7 | 9 |
| 5807-26.1 | 227 | 57 | 0.26 | 0.08 | 2.120 | 1.33 | 0.17978 | 0.82 | 2.122 | 1.33 | 0.17905 | 0.83 | 2489 | 27 | 2644 | 14 | 7 |

^206^Pb_c_ is the percentage of common ^206^Pb in total ^206^Pb estimated from measured ^204^Pb.

^207^Pb and ^206^Pb refer to total Pb, whereas ^207^Pb* and ^206^Pb* refer to radiogenic Pb after removal of common Pb as estimated from measured ^204^Pb.

Disc. is the discordance calculated as 100 {1 – (^206^Pb*/^238^U) / (exp [λ_238_ . t_(207Pb*/206Pb*)_] –1)}.

**Supplementary Table 2b: SHRIMP U-Pb zircon data for 8628-6006**

| Analysis Label | U (ppm) | Th (ppm) | ^232^Th/^238^U | ^206^Pb_c_ (%) | ^238^U/^206^Pb | ±1σ (%) | ^207^Pb/^206^Pb | ±1σ (%) | ^238^U/^206^Pb* (204 corr) | ±1σ (%) | ^207^Pb*/^206^Pb* (204 corr) | ±1σ (%) | ^238^U/^206^Pb* date (Ma) | ±1σ (Ma) | ^207^Pb*/^206^Pb* date (Ma) | ±1σ (Ma) | Disc (%) |
| --- | --- | --- | --- | --- | --- | --- | --- | --- | --- | --- | --- | --- | --- | --- | --- | --- | --- |
| 6006-1.1 | 149 | 58 | 0.41 | 1.02 | 7.289 | 1.50 | 0.07485 | 2.29 | 7.364 | 1.52 | 0.06643 | 4.05 | 821 | 12 | 820 | 85 | 0 |
| 6006-2.1 | 232 | 65 | 0.29 | 0.65 | 7.979 | 1.37 | 0.07505 | 3.42 | 8.031 | 1.39 | 0.06975 | 4.20 | 757 | 10 | 921 | 86 | 19 |
| 6006-2.2 | 593 | 151 | 0.26 | 0.25 | 11.385 | 1.20 | 0.06168 | 0.92 | 11.414 | 1.21 | 0.05966 | 1.44 | 541 | 6 | 591 | 31 | 9 |
| 6006-3.1 | 63 | 80 | 1.31 | 1.95 | 10.111 | 1.95 | 0.08954 | 7.80 | 10.312 | 2.05 | 0.07390 | 11.81 | 597 | 12 | 1039 | 238 | 45 |
| 6006-4.1 | 44 | 92 | 2.17 | 1.20 | 6.112 | 2.22 | 0.07554 | 2.54 | 6.186 | 2.26 | 0.06548 | 6.58 | 966 | 20 | 790 | 138 | -24 |
| 6006-5.1 | 43 | 79 | 1.90 | 1.64 | 8.107 | 2.27 | 0.09364 | 6.87 | 8.242 | 2.35 | 0.08042 | 10.30 | 738 | 16 | 1207 | 203 | 41 |
| 6006-6.1 | 228 | 77 | 0.35 | 0.00 | 6.851 | 2.73 | 0.07533 | 1.06 | 6.851 | 2.73 | 0.07533 | 1.06 | 878 | 22 | 1077 | 21 | 20 |
| 6006-6.2 | 622 | 151 | 0.25 | 0.11 | 11.033 | 1.20 | 0.06065 | 2.03 | 11.046 | 1.20 | 0.05973 | 2.17 | 559 | 6 | 594 | 47 | 6 |
| 6006-7.1 | 78 | 46 | 0.60 | 1.71 | 6.080 | 2.00 | 0.07903 | 1.83 | 6.186 | 2.04 | 0.06463 | 5.81 | 966 | 18 | 762 | 123 | -29 |
| 6006-8.1 | 262 | 296 | 1.16 | 0.28 | 6.209 | 1.35 | 0.07493 | 0.92 | 6.227 | 1.35 | 0.07257 | 1.41 | 960 | 12 | 1002 | 29 | 4 |
| 6006-9.1 | 113 | 52 | 0.47 | 0.44 | 6.951 | 1.76 | 0.07803 | 3.89 | 6.981 | 1.76 | 0.07441 | 4.56 | 863 | 14 | 1053 | 92 | 19 |
| 6006-9.2 | 801 | 205 | 0.26 | 0.21 | 11.051 | 1.18 | 0.06192 | 0.79 | 11.075 | 1.18 | 0.06019 | 1.18 | 557 | 6 | 610 | 25 | 9 |
| 6006-10.1 | 131 | 85 | 0.67 | 0.50 | 6.513 | 2.74 | 0.07652 | 1.51 | 6.546 | 2.75 | 0.07234 | 2.61 | 916 | 23 | 995 | 53 | 9 |
| 6006-11.1 | 448 | 78 | 0.18 | 0.50 | 9.151 | 1.24 | 0.06771 | 1.63 | 9.197 | 1.25 | 0.06364 | 2.28 | 665 | 8 | 730 | 48 | 9 |
| 6006-11.2 | 1811 | 323 | 0.18 | 0.11 | 11.057 | 1.12 | 0.06014 | 1.09 | 11.069 | 1.12 | 0.05921 | 1.18 | 558 | 6 | 575 | 26 | 3 |
| 6006-12.1 | 43 | 69 | 1.63 | 0.43 | 6.087 | 4.17 | 0.08768 | 7.31 | 6.113 | 4.18 | 0.08411 | 8.10 | 977 | 38 | 1295 | 158 | 26 |
| 6006-13.1 | 153 | 75 | 0.50 | 0.84 | 6.353 | 1.53 | 0.07997 | 2.69 | 6.407 | 1.55 | 0.07303 | 3.82 | 935 | 13 | 1015 | 77 | 8 |
| 6006-14.1 | 1165 | 243 | 0.22 | 1.76 | 10.817 | 1.15 | 0.07653 | 1.23 | 11.011 | 1.16 | 0.06227 | 2.52 | 560 | 6 | 683 | 54 | 19 |
| 6006-15.1 | 106 | 147 | 1.43 | 1.48 | 6.504 | 1.68 | 0.07965 | 1.62 | 6.601 | 1.71 | 0.06733 | 4.58 | 909 | 15 | 848 | 95 | -8 |
| 6006-16.1 | 47 | 85 | 1.87 | 5.35 | 6.265 | 2.25 | 0.09617 | 7.12 | 6.619 | 2.47 | 0.05067 | 22.22 | 907 | 21 | 226 | 514 | -324 |
| 6006-17.1 | 149 | 70 | 0.48 | 1.22 | 6.840 | 1.55 | 0.07778 | 3.04 | 6.924 | 1.58 | 0.06766 | 4.90 | 870 | 13 | 858 | 102 | -1 |
| 6006-18.1 | 98 | 88 | 0.93 | 2.25 | 10.422 | 1.75 | 0.07714 | 7.88 | 10.662 | 1.85 | 0.05880 | 13.41 | 578 | 10 | 560 | 292 | -3 |
| 6006-19.1 | 46 | 55 | 1.24 | 2.51 | 7.068 | 2.31 | 0.08215 | 2.66 | 7.249 | 2.43 | 0.06126 | 10.74 | 833 | 19 | 648 | 231 | -30 |
| 6006-20.1 | 55 | 106 | 1.99 | 4.29 | 6.686 | 2.14 | 0.08663 | 2.30 | 6.985 | 2.32 | 0.05027 | 15.40 | 862 | 19 | 207 | 357 | -338 |

^206^Pb_c_ is the percentage of common ^206^Pb in total ^206^Pb estimated from measured ^204^Pb.

^207^Pb and ^206^Pb refer to total Pb, whereas ^207^Pb* and ^206^Pb* refer to radiogenic Pb after removal of common Pb as estimated from measured ^204^Pb.

Disc. is the discordance calculated as 100 {1 – (^206^Pb*/^238^U) / (exp [λ_238_ . t_(207Pb*/206Pb*)_] –1)}.
